# Suspected Isolated Cardiac Sarcoidosis Initially Presenting as Sustained Ventricular Tachycardia

**DOI:** 10.7759/cureus.38974

**Published:** 2023-05-13

**Authors:** Abu Sufian

**Affiliations:** 1 Oncology, Aberdeen Royal Infirmary, Aberdeen, GBR

**Keywords:** palpitations, ventricular tachycardia, arrhythmia, cardiac sarcoidosis, sarcoidosis

## Abstract

A 65-year-old man was found to have suspected isolated cardiac sarcoidosis on a positron emission tomography (PET) scan to investigate the cause of sustained monomorphic tachycardia. The patient had episodes of palpitations 12 months prior to this admission, but no cause was ever discovered. Cardiac magnetic resonance (CMR) imaging revealed severe hypokinesis of the inferior segments of the left ventricle, which prompted a subsequent 18F-fluorodeoxyglucose (18F-FDG) PET/CT. The findings were consistent with potential isolated cardiac sarcoidosis as a cause of the fibrosis seen in the left ventricle. As such, the patient was commenced on immunosuppressive therapy and remains well to this day after being fitted with an implantable cardioverter defibrillator (ICD). Isolated cardiac sarcoidosis is a rare phenomenon but remains a diagnostic and therapeutic challenge for clinicians. We report a case of how isolated cardiac sarcoidosis can present as a cause of ventricular tachycardia.

## Introduction

Sarcoidosis is a multi-organ disease defined by non-caseating granulomas involving mostly the lungs and intra-thoracic lymph nodes. The development of sarcoidosis is poorly defined but is often cited to be a disease linked to a heightened immune response to an environmental antigen. More specifically, recent studies have postulated the key role of T-helper cells, especially that of Th17 cells, in modulating an “autoimmune” response observed in patients with sarcoidosis [1].

Affecting around seven people per 100,00, the disease commonly manifests in the lungs, and patients typically complain of dry cough and shortness of breath, often associated with cutaneous manifestations and painful joints. Sarcoidosis can mimic symptoms that are representative of certain cancers, especially lymphoma [2]. Patients presenting with night sweats, involuntary weight loss, and general fatigue undergo investigations primarily to exclude lymphoma [3]. The homogeneity between the symptoms of sarcoidosis and certain cancers and the rarity of sarcoidosis is precisely what makes it a diagnosis of exclusion.

Rarer still is a form of sarcoidosis affecting the myocardium; cardiac sarcoidosis. The variability in the initial presentation, ranging from benign ECG abnormalities to cardiac arrest, poses a diagnostic dilemma for clinicians. There is no universal investigative pathway to reach a diagnosis of cardiac sarcoidosis. Once a diagnosis is established, there is also a discrepancy with regard to the most optimal treatment.

## Case presentation

A 65-year-old male, who was otherwise fit and well, was admitted to the emergency department with palpitations. He has a family history of ischaemic heart disease and takes a statin. Prior to this admission, he presented with palpitations and chest pain 12 months earlier, which necessitated a follow-up with Cardiology. A Holter monitor revealed a first-degree heart block, atrial tachycardia, and short runs of ectopics.

During this hospitalization, he had a heart rate of 160 beats per minute, and ECG demonstrated a wide-complex tachycardia, for which he was treated with once-only bisoprolol 2.5 mg. Following the Cardiology consultation, the patient was transferred to the Cardiology department to undergo further investigations. Initial workup revealed cardiac enzymes, thyroid function, haematinics, immunoglobulins, ceruloplasmin, and copper to be within normal parameters. A negative quantiferon test to rule out TB was also performed.

As part of the workup, the patient underwent an echocardiogram, which revealed an overall preserved LV systolic function. However, hypokinesis of the basal inferoseptum and basal inferior wall was noted. A coronary angiogram revealed some mild atheroma in the left anterior descending (LAD) artery; otherwise, there was no obstructive disease. A cardiac MRI demonstrated an LV systolic function that was below normal in keeping with age and gender. During the late gadolinium portion of the study, marked mid-wall fibrosis of the basal inferoseptal and basal inferior segments of the LV was demonstrated. As such, there was marked hypokinesis in those areas, which was representative of a potential non-ischaemic cardiomyopathy diagnosis.

**Figure 1:**
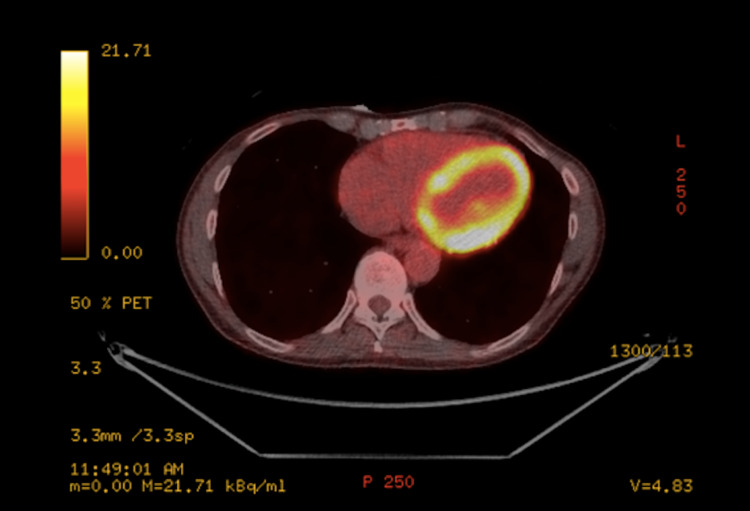
18F-FDG PET at baseline demonstrates increased uptake in the lateral and inferior walls of the left ventricle 18F-FDG PET: 18F-fluorodeoxyglucose positron emission tomography

18F-fluorodeoxyglucose (18F-FDG) positron emission tomography (PET)/computed tomography (CT) was subsequently conducted, demonstrating intense uptake of 18F-FDG within the lateral and inferolateral walls of the mid-left ventricle in addition to the basal inferior wall of the left ventricle. Importantly, there was moderate accumulation in the hilar lymph nodes bilaterally and a small right para-tracheal node. In combination, these findings were increasingly suspicious for sarcoidosis (Figure 1).

Following a respiratory review, a CT thorax abdomen pelvis (CT TAP) with contrast was performed to determine if lymph node biopsies were required. There were no significant changes to the appearance of the lungs compared to previous imaging done back on August 25, 2020. A nodular abnormality on the right upper lobe was unchanged and there was no evidence of significant lymphadenopathy in the mediastinal, hilar, or axillary regions. An endobronchial biopsy and bronchial washings from the right lower lobe and right upper lobe, respectively, were yielded from a bronchoscopy. The histological report found no evidence of granulomatous disease nor was there any cytological evidence of malignancy.

The patient’s long-term management was subjected to extensive discussion between cardiology and respiratory multidisciplinary meetings, given the increased risk of sudden death from ventricular arrhythmia. It was deemed clinically appropriate to implant an ICD to terminate any further ventricular tachycardias in the future. Prednisolone was commenced on discharge, and methotrexate was started a month later.

## Discussion

Clinicians face a diagnostic challenge due to the diverse clinical manifestations of cardiac sarcoidosis. The limited understanding of this condition creates a significant gap, and currently, there is no conclusive diagnostic test available to identify the disease.

In order to understand our patient's course, it is important to ascertain that cardiac sarcoidosis is defined by the granulomatous infiltration of cardiac tissue. In the case of our patient, there was no demonstrable evidence to confirm cardiac sarcoidosis owing to the lack of an endomyocardial biopsy. However, given the positive radiological findings in combination with the clinical presentation, the consensus among the multidisciplinary team (MDT) was to treat it as isolated cardiac sarcoidosis. Understandably, the lack of histological findings could be interpreted as a reason to suggest an inflammatory process - other than sarcoidosis - was responsible for the “hot” myocardium. 

Part of the complexity in diagnosing cardiac sarcoidosis lies in the lack of a gold standard protocol. The Japanese Ministry of Health and Welfare, revised in 2006, are a set of guidelines commonly referred to in the diagnostic workup of cardiac sarcoidosis [4]. Importantly, the revised version does not instruct the use of 18F-FDG PET/CT or attaining positive biopsies (either extra-cardiac or cardiac) in diagnosing cardiac sarcoidosis.

The Heart Rhythm Society recently published a working criterion to diagnose and manage arrhythmias associated with cardiac sarcoidosis [5]. More specifically, there is a requirement that a histological diagnosis of cardiac sarcoidosis requires the presence of non-caseating epithelioid granulomas from an endomyocardial biopsy. However, in our case, an endomyocardial biopsy was neither feasible due to the increased risk of complications from the procedure itself, nor offered at our facility. It is worth noting that an endomyocardial biopsy possesses a low sensitivity, particularly because of the sporadic pattern of disease, which limits its utility in diagnosing cardiac sarcoidosis. In addition, the safety profile of the procedure has led to an overall reduction in its use, particularly due to an increased risk of cardiac tamponade and stroke [6].

The next route to diagnosing cardiac sarcoidosis is to affirm histological extra-cardiac sarcoidosis alongside the presence of other cardiac abnormalities. This was unlikely in our case, as there were no respiratory or systemic symptoms to point toward sarcoidosis. Additionally, the negative result of the endobronchial biopsy ruled out the possibility of making a clinical diagnosis of cardiac sarcoidosis.

**Figure 2:**
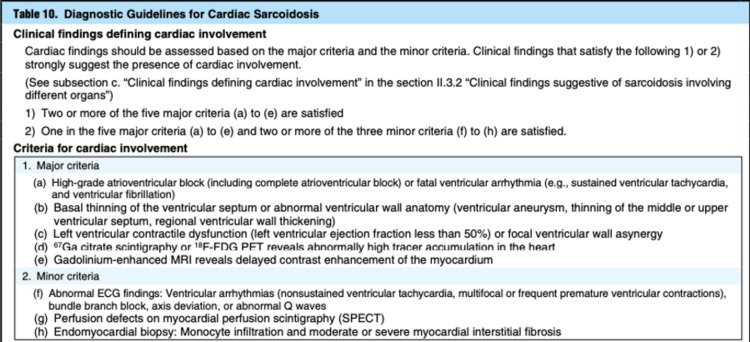
Japan Circulation Society (JCS) 2016 guidelines for diagnosing cardiac sarcoidosis

As such, according to the updated guidelines of the Japan Circulation Society (JCS), a diagnosis of isolated cardiac sarcoidosis was suspected. In our case, the patient had satisfied two of the five major criteria to ascertain a probable diagnosis of isolated cardiac sarcoidosis (Figure 2). An ICD was fitted following an MDT discussion between cardiac electrophysiology and respiratory.

**Figure 3:**
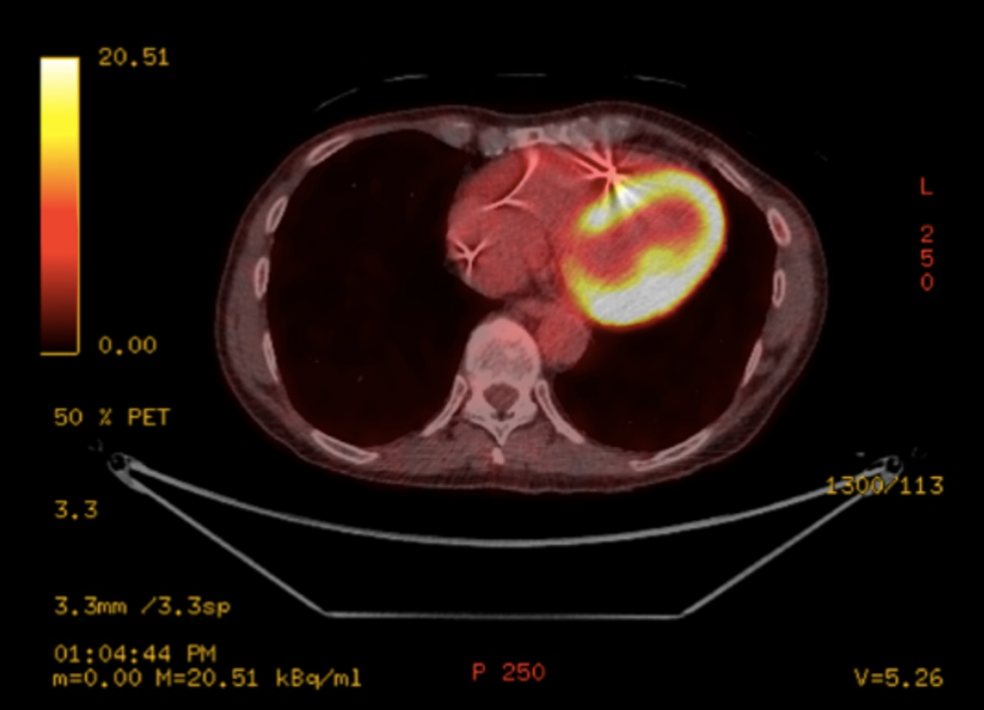
Reduced uptake of 18F-FDG in repeat PET/CT three months following discharge 18F-FDG: 18F-fluorodeoxyglucose; PET/CT: positron emission tomography/computed tomography

The patient was discharged on prednisolone 50 mg OD and advised to reduce the dosage every fortnight until a maintenance dose of 10 mg OD was reached. Additionally, the patient was commenced on methotrexate a month after starting steroid therapy. Figure 3 shows the reduced uptake of 18F-FDG in a repeat PET/CT three months after discharge.

## Conclusions

Part of the challenge of identifying isolated cardiac sarcoidosis is the lack of extra-cardiac involvement, whereas cardiac sarcoidosis represents systemic sarcoidosis commonly affecting the lungs. Our case clearly demonstrates the need for clinicians to possess a low threshold for isolated cardiac sarcoidosis in the context of ventricular tachycardia.
